# The Effect of Commuting Patterns on HIV Care Attendance Among Men Who Have Sex With Men (MSM) in Atlanta, Georgia

**DOI:** 10.2196/publichealth.4525

**Published:** 2015-08-24

**Authors:** Sharoda Dasgupta, Michael R Kramer, Eli S Rosenberg, Travis H Sanchez, Landon Reed, Patrick S Sullivan

**Affiliations:** ^1^ Emory University Atlanta, GA United States; ^2^ Atlanta Regional Commission Atlanta, GA United States

**Keywords:** men who have sex with men, HIV care, commuting patterns, public transportation

## Abstract

**Background:**

Travel-related barriers to human immunodeficiency virus (HIV) care, such as commute time and mode of transportation, have been reported in the United States.

**Objective:**

The objective of the study was to investigate the association between public transportation use and HIV care attendance among a convenience sample of Atlanta-based, HIV-positive men who have sex with men (MSM), evaluate differences across regions of residence, and estimate the relationship between travel distance and time by mode of transportation taken to attend appointments.

**Methods:**

We used Poisson regression to estimate the association between use of public transportation to attend HIV-related medical visits and frequency of care attendance over the previous 12 months. The relationship between travel distance and commute time was estimated using linear regression. Kriging was used to interpolate commute time to visually examine geographic differences in commuting patterns in relation to access to public transportation and population-based estimates of household vehicle ownership.

**Results:**

Using public transportation was associated with lower rates of HIV care attendance compared to using private transportation, but only in south Atlanta (south: aRR: 0.75, 95% CI 0.56, 1.0, north: aRR: 0.90, 95% CI 0.71, 1.1). Participants living in south Atlanta were more likely to have longer commute times associated with attending HIV visits, have greater access to public transportation, and may live in areas with low vehicle ownership. A majority of attended HIV providers were located in north and central Atlanta, despite there being participants living all across the city. Estimated commute times per mile traveled were three times as high among public transit users compared to private transportation users.

**Conclusions:**

Improving local public transit and implementing use of mobile clinics could help address travel-related barriers to HIV care.

## Introduction

### Importance of Regular Human Immunodeficiency Virus Care Engagement

Men who have sex with men (MSM) accounted for 67% of all new human immunodeficiency virus (HIV) infections in the United States in 2012, despite accounting for only 3% of the population [1,2]. Regular medical care utilization among HIV patients is important in maintaining viral load suppression, reducing transmission to others [3-5], and improving survival over time [5,6]. Among newly diagnosed cases in Georgia in 2011, an estimated 46% were regularly engaged in HIV care and 45% had achieved viral suppression within 15 months of diagnosis [7]. Similar estimates in HIV care engagement were observed among a large cohort of MSM in Atlanta [6].

### Transportation Factors as Barriers to Human Immunodeficiency Virus Care

Transportation-related factors, such as travel distance and commute time, have been reported as substantial barriers to general medical care and attending HIV appointments [8-15]. Transportation assistance was reported as an unmet need among 16% of those who needed it in a cross-sectional, nationally representative study of HIV-positive individuals engaged in care [16]. Travel distance and mode are both important predictors of commute time [17] and can influence travel times differentially by neighborhood, depending on availability of public transportation, household vehicle ownership, and traffic congestion patterns [18,19].

Compared to traveling by car, using public transportation is often associated with longer commute times and reduced convenience and flexibility in travel [17,20]. This is especially important in cities like Atlanta with limited public transportation and a strong dependence on travel by car [21]. Longer commute times can be a deterrent to attending care visits, especially with competing household and job responsibilities [8]. In Atlanta, the Metropolitan Atlanta Rapid Transit Authority (MARTA) has been the primary source of public transportation infrastructure for both bus and rail since the 1970s. MARTA, as well as other transit systems in the metro area, serves mostly urban areas in the city [22,23].

Atlanta has historically been a highly segregated city with respect to race and income [24], with differential access to public transportation. A general north-south pattern exists; with predominantly lower income, black neighborhoods in south Atlanta and mostly white neighborhoods in north Atlanta. Neighborhood contextual factors, such as availability of resources and socioeconomic deprivation, have been shown to be associated with negative physical and mental health outcomes [25-27]. Our study objectives were two-fold. First, we investigated whether public transportation use is associated with HIV care attendance and whether the association varies by region of residence (north vs south) in Atlanta. Second, because taking public transportation can strongly affect commute times, we also estimated the relationship between travel distance and commute time by mode of transportation. Identifying areas where travel-related factors might be barriers to HIV care can be beneficial in planning targeted structural interventions to improve health care utilization.

## Methods

### Study Methodology

#### Recruitment

The Engage Study, a cross-sectional study of self-identifying HIV-positive MSM, was designed to investigate structural and psychosocial barriers to HIV care among MSM living in the Atlanta area. A convenience sample of men was recruited from October 2012 to June 2013 from two sources: (1) based on participation in previously conducted Atlanta-based studies on HIV, and (2) from Facebook. Men who previously participated in the Atlanta-based studies and had a known positive HIV test were contacted by phone and email for recruitment. Individuals interested in participation were then sent a Web link to the Internet eligibility screener by email. Participants from Facebook were recruited based on banner advertisements targeting men who were interested in other men and lived within 50 miles of Atlanta. Those who clicked on the banner advertisements were directed to the Internet eligibility screener.

Individuals were eligible for participation if they reported being 18 years of age or older, being told they were HIV-positive by a health care provider, having sex with at least one other man in their lifetime, and living in the Atlanta area. All consenting participants were directed from the Internet eligibility screener to the questionnaire administered using a Health Insurance Portability and Accountability Act (HIPAA)-compliant Internet survey software platform, SurveyGizmo (Boulder, CO) [28]. The Emory University Institutional Review Board approved the study protocol (approval number: IRB00060430). More details on study methodology have been previously described [29].

#### Measures

The Web-based questionnaire collected information on demographic characteristics, potential structural (eg, public transportation use, health insurance status) and psychosocial barriers (eg, perceived HIV-related stigma, disclosure of HIV status, self-perceived community perceptions of HIV) to HIV care engagement, and characteristics related to HIV care experiences (eg, number of attended care appointments with the most recently attended HIV provider). We also collected information about home address at the time of the interview and location of the last HIV care provider, which was used to estimate travel distance and commute time to attend care visits. We geocoded all addresses using ArcGIS 10.2 (Redlands, CA). Participants were also asked about mode of transportation used to regularly attend care. Those reporting normally traveling by train, bus, or foot were considered to be public transportation users; otherwise, they were considered private transportation users.

To estimate travel distance and commute time between participant residence and last attended HIV provider, we used the Google Maps Directions application programming interface. Distance and commute time were calculated for each pair of origin-destination points (ie, residence and provider locations) based on the most optimal route chosen by Google maps. Travel parameters were calculated separately for those who took public transportation versus those who did not. Those who did not take public transportation were assumed to travel by car. All travel parameters were calculated assuming a departure day and time of Friday, March 7, 2014 at 10:00 AM. Latitude-longitude coordinates for residence were anonymized before entered into Google Maps to protect confidentiality of participants.

**Figure 1:**
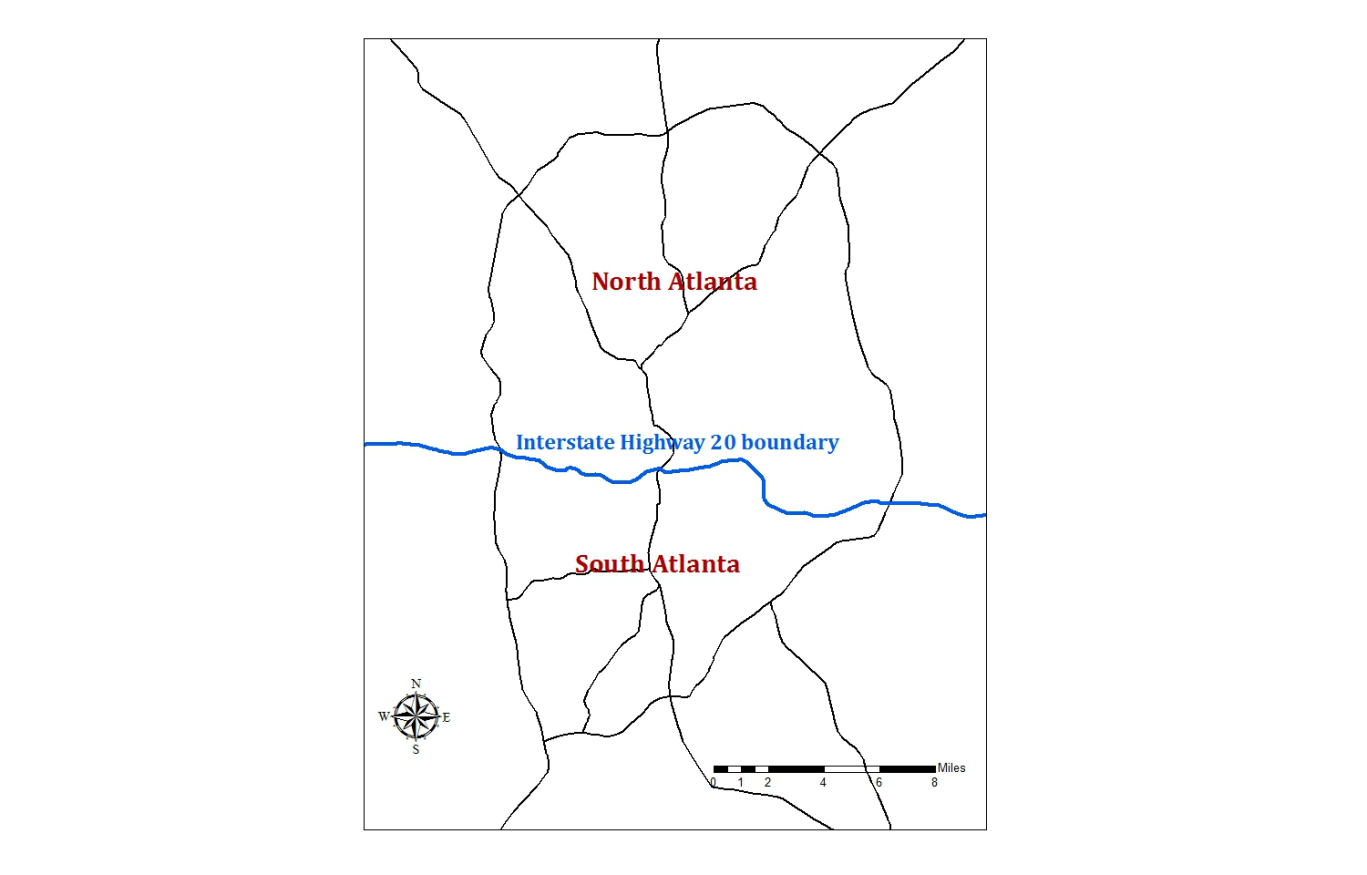
Geographic boundaries defining regions of residence used in analyses.

Since our research objectives focused on assessing differences in effect estimates across region of residence, we stratified the results by residence in north versus south Atlanta. Interstate highway 20 served as a coarse boundary for these regions, as it is often used to distinguish between areas of differing socioeconomic status, such as racial composition and average household income [24] (Figure 1 shows this).

We obtained information on household vehicle access, which we used as a proxy for vehicle ownership, from the US Census Bureau [30] and data on availability of public transportation bus and train routes from the Atlanta Regional Commission [31].

### Analytic Methods

#### Descriptive Statistics

Descriptive statistics were computed for transportation-related factors, demographic characteristics, and the number of attended HIV care appointments in the previous 12 months, overall and by region of residence (north vs south). We reported medians and interquartile ranges (IQR) for continuous variables, and counts and frequencies for categorical variables. Wilcoxon-Mann-Whitney tests were used to evaluate differences in continuous variables across region of residence. For categorical variables, differences were assessed using the Mantel-Haenszel chi-square test.

#### Modeling

##### Public Transportation and Human Immunodeficiency Virus Care Attendance

A Poisson regression model estimated the rate of attended HIV care-related visits with the most recent provider in the past 12 months (using attended HIV care appointment counts) as a function of whether or not public transportation was taken to attend care, and examined whether the association was modified by region of residence in the city. The offset variable represented eligible days to receive HIV care in the past 12 months, and was coded as the natural log of 365 days unless date of diagnosis was less than a year before the survey was completed, in which case it represented the number of days between the date of the survey and date of diagnosis.

Race was considered an important confounding variable, and thus was included, *a priori*, in the final, multivariable model. Mode of transportation, region of residence, and the interaction between the two variables were also retained in the final model, since they were the primary explanatory variables of interest. For other covariates, bivariate associations with the outcome of interest were assessed, and variables with *P* values of less than 0.1 were eligible for possible inclusion in the final multivariable model. Except for the variables included *a priori* in the analysis, backward selection was used to determine which variables should be retained in the final model, using a cutoff of *P*<.05. The multivariable Poisson model was built using SAS 9.3 (Cary, NC).

In a post-hoc analysis, we examined spatial relationships between estimated commute time, the network of available public transportation routes in Atlanta, and areas with low household vehicle ownership. Using ArcGIS 10.2, we utilized kriging to interpolate commute time associated with traveling to the last attended HIV provider. Because we did not ask participants directly about individual household vehicle ownership, we used US Census data as a marker for study areas with poor access to a vehicle. We defined vehicle ownership as having access to one or more vehicles in the household (a proxy for ownership); census tracts with < 87% household vehicle access were considered areas of low ownership. We based this cutoff on results from the 2009 National Household Travel Survey, which estimated that approximately 13% of US households were without access to a vehicle in large urban areas [32]. Using GeoDa (Tempe, AZ), local Moran’s I statistics with significance testing (alpha = .05) evaluated local spatial autocorrelation to identify clusters of low vehicle ownership geographically.

##### Travel Distance and Commute Time

We used a linear regression model to describe the relationship between travel distance and commute time, stratified by mode of transportation used to attend appointments. For each mode of travel, the intercept represented initial investment in time; the slope provided information on the increase in commute time for each mile traveled. No other covariates were of interest, and therefore, were not included in the final model.

## Results

### Descriptive Statistics

A total of 213 eligible MSM participated; 205/213 (96.2%) participants reported ever receiving HIV care, among which 184/205 (89.7%) reported valid location data on home address and last HIV provider to enable calculation of road distance and commute time between the two. A total of 178/184 (96.7%) respondents who traveled less than 100 miles and lived within 50 miles from the center of Atlanta were used in the final analysis dataset.

The median age of participants was 34 years old, over half of participants reported an annual household income of less than US $20,000, and about two-thirds identified as black/African American race (Table 1). Participants attended a median of 3 appointments with their most recent HIV care provider in the previous 12 months, and about a third of participants reported missing at least one appointment. Overall, (72/178) 40% reported using some form of public transportation to attend care; median commute time was 22 minutes and median travel distance was about 9 miles.  

Participants living in south Atlanta were significantly more likely to report black race (*P*<.001), have lower annual household income (*P*=.04), and not have health insurance at the time of the survey (*P*=.03). Greater reported use of public transportation (*P*=.002), travel distance (*P*=.003), and commute times (*P*<.001) associated with attending HIV care visits were observed in south Atlanta, compared to north Atlanta. Participants in south Atlanta were also more likely to live in census tracts with low vehicle ownership, but this difference was not statistically significant (*P*=.05).

**Table 1:** Demographic characteristics reported among a convenience sample of HIV-positive MSM linked to care in Atlanta, Georgia, 2012-2013.

		Overall^a^			North Atlanta^a^			South Atlanta^a^		
Demographic characteristics		n	%	Mean visits	n	%	Mean visits	n	%	Mean visits
**People living in tracts with low household vehicle ownership**										
	< 87%	64	36	3.5	41	33	3.5	23	44	3.3
	> 87%	114	64	3.2	85	67	3.1	29	56	3.6
**Taking public transit (bus, train, foot) to attend care visits** ^b^										
	Yes	72	40	3.4	42	33	3.4	30	58	3.3
	No	106	60	3.3	84	67	3.1	22	42	3.8
**Age in years**										
	< 35 years	91	51	2.9	65	52	3.0	26	50	2.7
	> 35 years	87	49	3.7	61	48	3.4	26	50	4.3
**Race** ^b^										
	White	60	34	3.3	52	41	3.1	8	15	4.5
	Black/African American	107	60	3.4	66	52	3.4	41	79	3.3
**Education**										
	High school or less	32	18	3.2	20	16	2.9	12	23	3.6
	At least some college	144	81	3.4	104	83	3.3	40	77	3.5
**Annual household income** ^b^ **(US)**										
	< $20,000	93	52	3.3	61	48	3.2	32	62	3.6
	> $20,000	79	44	3.2	62	49	3.1	17	33	3.6
**Current health insurance status** ^b^										
	Yes	102	57	3.3	78	62	3.2	24	46	3.6
	No	74	42	3.3	46	37	3.2	28	54	3.4

^a^ Numbers may not sum to total because of missing values. Percentages may not add up to 100 due to rounding.

^b^ Statistically significant differences observed across region of residence, alpha =.05.

### Modeling

#### Public Transportation and Human Immunodeficiency Virus Care Attendance

**Figure 2:**
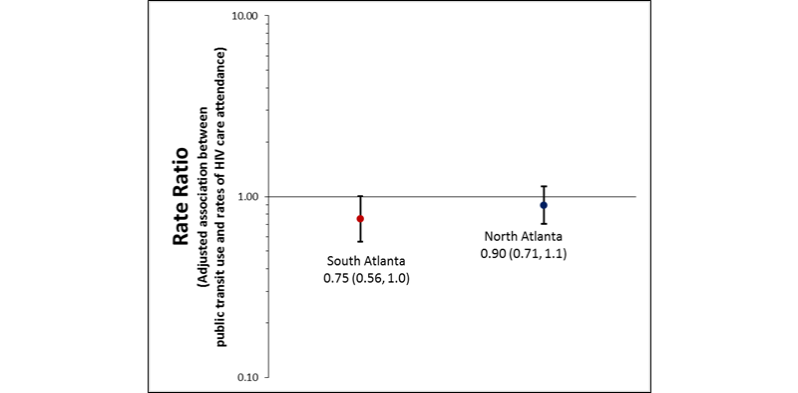
Adjusted association between public transportation use and rates of HIV care attendance in the past 12 months, by region of residence, among a convenience sample of HIV-positive MSM linked to care, Atlanta, Georgia, 2012-2013. Final multivariable model controls for race, annual household income, and health insurance status reported at the time of interview.

Of those living south Atlanta, the adjusted rate of HIV care attendance was 25% lower among those who took public transportation to attend care visits, compared to those who took private transportation (aRR: 0.75, 95% CI 0.56, 1.0; Figure 2 shows this). No significant association was observed among those living in north Atlanta (aRR: 0.90, 95% CI 0.71, 1.1). The multivariable model adjusted for race, annual household income, and health insurance status reported at the time of the interview. Although the interaction between region of residence and use of public transportation was not significant, stratified results are presented because we hypothesize that factors related to socioeconomic status (SES), such as reasons for taking public transportation to attend visits, might vary depending on region of residence

**Figure 3:**
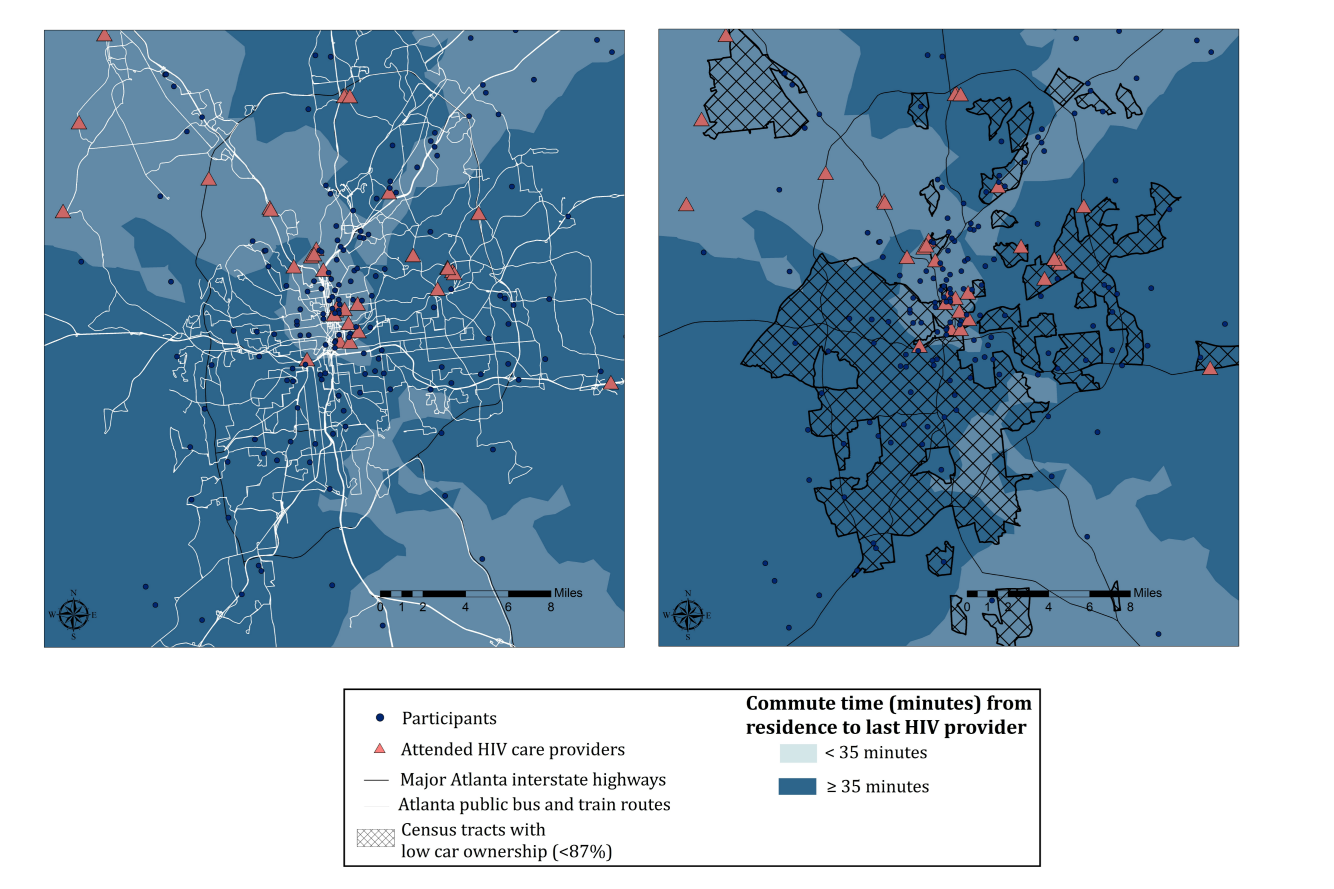
Relationships between interpolated commute time and network of public transportation routes (left), and interpolated commute time and low car ownership (right), among a convenience sample of HIV-positive MSM linked to care, Atlanta, Georgia, 2012-2013. Locations of HIV providers attended by participants are denoted in pink triangles, and locations of participant residences, which have been anonymized, are represented by dark blue dots.

To explore this hypothesis, we examined interpolated commute times across urban areas of Atlanta with respect to census tracts with low car ownership and the network of available public transportation in the city (Figure 3 shows this). Interpolated commute times greater than the overall mean (34 minutes) were observed in much of south Atlanta. A majority of attended HIV providers were also located in north or central Atlanta, which may have driven longer commute times observed for participants living in south Atlanta.

Further, positive spatial autocorrelation was observed in a cluster of census tracts with low car ownership in south Atlanta. Within the auxiliary interstate highway 285 (often used as a boundary for urban vs suburban/rural areas of Atlanta), there are 6.6 miles of available public transportation per 1000 population in south Atlanta and 4.4 miles of available transit per 1000 population in north Atlanta.

#### Travel Distance and Commute Time

**Figure 4:**
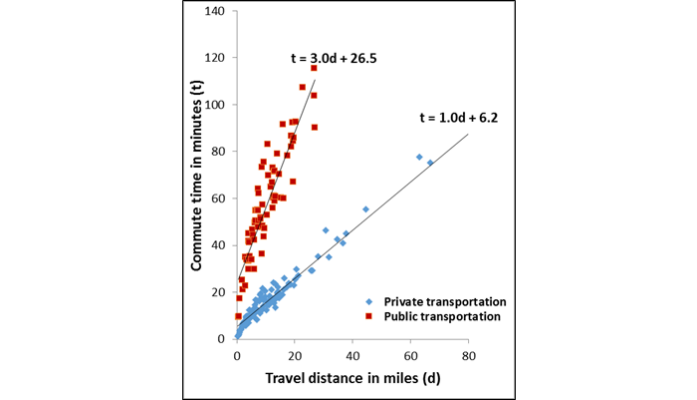
Linear relationship between travel distance (miles) and commute time (minutes) to HIV care appointments, stratified by use of public transportation to attend HIV care appointments, among a convenience sample of HIV-positive MSM linked to care, Atlanta, Georgia, 2012-2013. In each of the estimated regression equations below, t represents commute time in minutes, d represents travel distance in miles, and the intercept represents the estimated time investment made in taking a specified mode of travel to attend HIV appointments.

The modeled estimates showed that the relationship between travel distance and commute time varied by mode of transportation taken to attend HIV care visits (Table 2 and Figure 4 show this). The model explained 93% of the variance of the data around the estimated regression equation. The estimated initial time investment associated with commuting was over 4 times higher among public transportation users (27 minutes) compared to private transportation users (6 minutes). Among those who took private transportation, each mile of travel resulted in an additional minute of commute time; by contrast, the rate of increase in commute time per mile traveled was 3 times as high among those who took public transportation. Estimated commute times were consistently longer for public transportation users; differences in commute times for key distance values are provided in Table 2.

**Table 2:** Modeled estimates for initial time investment, rate of increase in commute time per mile traveled, and differences in overall commute time (for key distances) by mode of transportation taken to attend HIV care visits among a convenience sample of HIV-positive MSM linked to care in Atlanta, Georgia, 2012-2013.

Mode of transportation	Initial investment (minutes)	∆ Commute time per mile traveled (minutes)	Modeled commute time (minutes) for miles traveled			
			1 mile	5 miles	10 miles	15 miles
Public^a^	26.5 (23.6, 29.5)	3.0 (2.8, 3.3)	29.5	41.7	56.9	72.0
Private^a^	6.2 (4.5, 7.9)	1.0 (0.9, 1.1)	7.2	11.4	16.5	21.7

^a^ Change in modeled commute times by mode of transportation for key travel distances, miles, listed in table: 22.3 minutes, 30.3 minutes, 40.3 minutes, 50.4 minutes.

## Discussion

### Principal Findings

In this study, we investigated commuting patterns related to attending HIV care visits, a topic which has not been extensively explored among HIV-positive MSM in Atlanta. Among those living in south Atlanta, using public transportation was associated with lower rates of HIV care attendance, compared to using private transportation. Participants in south Atlanta had greater access to public transportation (miles/1000 population), but traveled longer and further to attend HIV appointments and may be more likely to live in areas with low vehicle ownership. Both initial time investment and rate of increase in commute time per mile traveled to attend HIV visits were significantly higher among those who took public transportation, compared to those who did not.

Although not statistically significant, geographic differences in the association between public transportation use and care attendance could signify that transportation was more of a barrier to attending HIV care visits in south Atlanta. Because the sample size was limited, a larger study may have detected statistically significant differences in the association. We hypothesize that if the geographic differences in effect estimates exist, they may be driven by factors related to SES, such as differing reasons for taking public transportation, or differences in availability of medical resources around the metro area.

Although using public transportation is often associated with longer and more variable commute times and reduced flexibility in travel, there may be many reasons why public transportation is preferred, including: (1) concerns related to traffic congestion and pollution, (2) cost reductions associated with traveling by car, (3) convenience, if residence is in an urban area with access to public transit and limited space for private vehicle parking, and (4) not having another means of travel [17,20,33,34]. Out of these four reasons, the first three are related to convenience, or choice to take public transportation, while the fourth is associated with necessity because of lack of vehicle ownership. Because levels of household vehicle ownership may be higher in north versus south Atlanta, we hypothesize that those living in south Atlanta might be more likely to take public transportation out of necessity, and people living in north Atlanta might choose to take public transportation out of convenience. Although we did not directly measure reasons for taking certain modes of transit in the present study, exploring differences in reasons for taking public transportation in the future may be helpful in understanding complex patterns and dynamics between travel and medical care utilization.

When we examined a combination of population-based transportation-related factors with the Engage Study data to get a clearer picture of reasons for taking public transportation across Atlanta, we found that south Atlanta had overlapping geographic areas of longer estimated commute times to attend HIV care visits, low car ownership, and greater access to public transportation. Historically, south Atlanta also has a majority black population and greater levels of poverty compared to other areas of Atlanta [24], and along with downtown Atlanta, also has a greater burden of HIV compared to other areas of the city [35]. National data also show disproportionately greater use of public transportation among minorities and individuals from low-income households, suggesting socioeconomic differences in travel behaviors [36]. Therefore, transportation-related barriers to HIV care may be more prevalent in economically disadvantaged communities in south Atlanta where there is a greater need for HIV medical care utilization.

Although reasons for taking public transportation are highly correlated with SES, controlling for census tract-level car ownership and individual-level race and income in this analysis did not explain the observed association between public transportation use and HIV care attendance in south Atlanta. Individual-level household vehicle ownership could have explained the association if the participants are not representative of their census tract of residence. However, information on individual-level vehicle ownership was not available. Alternatively, there may have been one or more unmeasured factors associated with neighborhood economic disadvantage and deprivation, which explain the differential results between north and south Atlanta, and this should be further explored.

Differences in the density of available medical resources may have also helped drive the geographic differences in effect estimates. In particular, there were very few attended HIV providers located in south Atlanta, compared to north Atlanta. This is consistent with another study, which found poorer spatial accessibility to HIV providers in south Atlanta, where HIV prevalence is high [37]. Having fewer available providers in an area where residents are potentially more reliant on public transit as a sole means of travel might amplify travel-related barriers. Exploring this idea further in future studies through focus groups may help elucidate important drivers of travel-related barriers to HIV care.

Although this is a hypothesis-generating study with exploratory objectives, the results justify exploring in the future whether travel-related barriers affect medical care attendance differentially by region of residence among Atlanta-based, HIV-positive MSM. Larger studies which collect information on individual car ownership and any unmeasured factors which could potentially explain the differential effect estimates would help inform whether interventions related to improving spatial access might be beneficial. For instance, if transportation did indeed differentially affect HIV care attendance, the use of mobile clinics, as well as expansion of public transportation networks and more frequently operating bus and train routes, could be helpful in mitigating travel-related barriers.

Mobile vehicles used for HIV testing have been accepted by patients both in and out of the United States [38-40], but have rarely been used to administer HIV care, despite such an option being suggested to reduce transportation and socioeconomic barriers to medical care [41]. Mobile clinics have been used to provide other types of medical care previously, and have been associated with improved health care utilization [42], and potentially, fewer visits to the emergency department [43], after implementation.

Improving public transportation connectivity to other parts of the city, where preferred HIV clinics may operate, is key to increasing mobility of lower income communities that may be less likely to own a vehicle. Increasing frequency of existing public bus and train routes may also cut down on commute times and improve the level of convenience associated with taking public transportation. However, expansion of public transit in Atlanta has continually been a contentious issue among the public [23,44]. The original plan for a public transit system was published in a 1961 report by the Atlanta Region Metropolitan Planning Commission, and included an expansive, 66 mile rail network and covered five counties in the metro area [21,22]. Unfortunately, the plan was not approved by voters and eventually resulted in the 48 mile rail and 91 route bus system that it is today [22]. Despite limited availability of funds to expand the current public transportation network, incorporating discussions about public health during transit planning would be helpful in serving communities, which may benefit from greater access to medical resources they may need.

### Limitations

There are several limitations to this study. First, the cross-sectional study design does not lend itself to making inferences on temporality or causality. Second, because a large proportion of participants were recruited on the Internet, HIV status was self-reported and could not be verified. However, we suspect little to no misclassification of HIV status because the study survey contained extensive questions about provider location and HIV care engagement. The primary outcome, number of attended HIV care visits in the past 12 months, was self-reported, and therefore, is subject to information bias. We hypothesize that the number of attended appointments might be overreported, but do not suspect that misclassification was differential with respect to travel parameters.

The relationship between travel-related factors and HIV care attendance may be confounded by level of disease progression, which should be incorporated in future analyses. Results were generated from a convenience sample, which may not be representative of all HIV-positive MSM living in Atlanta, limiting generalizability of results. Obtaining more information on mode of transportation used to attend visits, including whether the patient carpooled with someone or received a ride and reasons for taking certain modes of transit, would have added to the results. In addition, using population-based estimates of vehicle access as a proxy for household vehicle ownership for participants may not have been appropriate due to convenience sampling. Finally, home and provider locations are based on self-report and are subject to information bias.

### Conclusions

Using public transportation, compared to private transportation, may have been a barrier to HIV care among a sample of Atlanta-based, HIV-positive MSM living in south Atlanta. We hypothesize that reasons for taking public transportation and availability of HIV providers may differ across regions of residence in Atlanta, and, thus, could help explain the differences in the observed association by region. However, this hypothesis should be further explored in future studies.

The results from this analysis add to the current knowledge about travel and transportation-related barriers to HIV care, may inform the design of larger population-based studies which further explore potential neighborhood-level characteristics driving differences in travel-related barriers, and could provide guidance on potentially beneficial interventions which address gaps in care among Atlanta-based, HIV-positive MSM.

## Abbreviations

HIV: human immunodeficiency virus
IQR: interquartile ranges
MARTA: Metropolitan Atlanta Rapid Transit Authority
MSM: men who have sex with men
SES: socioeconomic status
